# Molecular cytogenetic analysis of some Picea species from Eurasia and East Asia

**DOI:** 10.18699/vjgb-26-74

**Published:** 2026-09

**Authors:** O.V. Goryachkina, E.D. Badaeva, A.B. Shcherban, E.N. Muratova

**Affiliations:** V.N. Sukachev Institute of Forest of the Siberian Branch of the Russian Academy of Sciences, Federal Research Center “Krasnoyarsk Science Center of the Siberian Branch of the Russian Academy of Sciences”, Krasnoyarsk, Russia; N.I. Vavilov Institute of General Genetics of the Russian Academy of Sciences, Moscow, Russia Engelhardt Institute of Molecular Biology of the Russian Academy of Sciences, Moscow, Russia; Institute of Cytology and Genetics of the Siberian Branch of the Russian Academy of Sciences, Novosibirsk, Russia; V.N. Sukachev Institute of Forest of the Siberian Branch of the Russian Academy of Sciences, Federal Research Center “Krasnoyarsk Science Center of the Siberian Branch of the Russian Academy of Sciences”, Krasnoyarsk, Russia

**Keywords:** chromosome, karyotype, FISH, 45S rDNA, 5S rDNA, DAPI staining, Picea, хромосома, кариотип, FISH, 45S рДНК, 5S рДНК, окрашивание DAPI, Picea

## Abstract

Karyotypes of seven Picea species distributed across Eurasia and East Asia were analyzed using fluorescence in situ hybridization (FISH) and DAPI fluorescent staining. For P. meyeri and P. purpurea, the 5S and 45S rDNA sites were mapped here for the first time, while karyotypes of P. abies, P. obovata, P. schrenkiana, P. crassifolia, and P. omorika were examined from new locations. All the species studied had a similar distribution of 5S rDNA loci on both arms of the large metacentric chromosome III: a more intense signal was observed in the middle of the long arm, partially overlapping with the 45S rDNA signal, and a minor 5S rDNA site was localized terminally on the short arm. The distribution of 5S rDNA loci varied in the karyotypes of the studied species: from 7 to 9 pairs of chromosomes carried 45S rDNA signals of varying intensity. Strong (major) signals were localized intercalarily and coincided with persistent secondary constrictions. Their number per haploid genome was as follows: 8 in P. omorika, 6 in P. abies and P. obovata, and 5 in P. schrenkiana, P. crassifolia, P. purpurea, and P. meyeri. We also detected several minor 45S rDNA sites on the Picea chromosomes that had not been previously described in the literature. Supernumerary (B) chromosomes found in some seedlings of P. obovata, P. meyeri, and P. purpurea did not carry 45S and 5S rDNA loci. Intensely fluorescent DAPI-positive bands were observed in the intercalary regions of the spruce chromosomes, and the centromere regions of certain chromosomes were also stained. Several constant DAPI-positive blocks were identified on chromosomes I, III, and X, with similar locations across all studied species. Fluorescence in situ hybridization with 45S and 5S rDNA probes in combination with DAPI staining allowed us to identify virtually all homologous chromosomes and compare karyotypes of different species. The intra- and interspecific karyotypic diversity in the genus Picea is discussed considering the distribution of 5S and 45S rDNA loci and DAPI signals.

## Abstract (ru)

Представлены результаты молекулярно-цитогенетического исследования семи видов Picea, распространенных на территории Евразии и Восточной Азии, методом флуоресцентной гибридизации in situ (FISH) и флуоресцентного окрашивания DAPI. Кластеры рибосомных генов 45S и 5S рРНК на хромосомах P. meyeri и P. purpurea картированы впервые, P. abies, P. obovata, P. schrenkiana, P. crassifolia, P. omorika изучались из новых местопроизрастаний. Все виды показали сходное распределение локусов 5S рДНК на обоих плечах крупной метацентрической хромосомы III: более интенсивный сигнал наблюдался на длинном плече, частично перекрываясь с сигналом 45S рДНК, минорный сайт 5S рДНК был локализован терминально на коротком плече. Сигналы 45S рДНК разной интенсивности были выявлены на 7–9 парах хромосом в кариотипах исследуемых видов. Яркие (мажорные) сигналы локализованы интеркалярно и совпадали с постоянными вторичными перетяжками. Их количество на гаплоидный геном составило: у P. omorika – 8, у P. abies и P. obovata – 6, у P. schrenkiana, P. crassifolia, P. purpurea и P. meyeri – 5. Выявлено также несколько минорных сайтов 45S рДНК на хромосомах Picea, не описанных ранее в литературе. Добавочные (В) хромосомы, обнаруженные у некоторых проростков P. obovata, P. meyeri и P. purpurea, не несли локусов рибосомных генов 45S и 5S рРНК. На хромосомах ели наблюдались интенсивно флуоресцирующие DAPI-позитивные полосы в интеркалярных районах, а у некоторых хромосом окрашивалась также область центромеры. На хромосомах I, III и X пар удалось выявить несколько постоянных позитивных DAPI-блоков, имеющих сходную локализацию у всех изученных видов. FISH с зондами 45S и 5S рДНК в сочетании с окрашиванием DAPI позволяет идентифицировать практически все гомологичные пары хромосом и провести сравнение кариотипов разных видов ели. Обсуждается внутри- и межвидовое кариотипическое разнообразие в роде Picea на основе распределения локусов 5S и 45S рДНК и рисунка DAPI.

## Introduction

The genus Picea A. Dietr. (Spruce) consists of 35–50 species; the number of species discriminated within a genus depends on the taxonomic system (Sukachev, 1928; Dallimore, Jackson, 1966; Bobrov, 1971; Liu T.S., 1982; Farjon, 1990, 2001; Lockwood et al., 2013). More than half Picea species grow in Siberia, Russian and foreign Far East, in the Pacific basin. They are among the main forest formers of the Northern Hemisphere and are of great practical importance. The spruce species exhibit significant morphological diversity, which might be due to their wide geographical range from temperate to subarctic regions.

Picea karyotypes consist of 12 chromosome pairs (2n = 24): eight pairs (I–VIII) are long metacentrics, two pairs (X, XI) are short metacentrics, and two pairs (IX, XII) are short submetacentric chromosomes (Liu Y.H., Li, 1985; Hizume, 1988; Li et al., 2001; Nkongolo, Mehes-Smith, 2012) The chromosomes of I–VIII pairs are similar in length and arm ratio, so it is not always possible to define pairs of homologs using traditional staining methods. Supernumerary B chromosomes, more often of the metacentric type, have been found in 21 Picea species (Muratova et al., 2023).

Fluorescence in situ hybridization (FISH) has great prospects in plant karyotype analysis, allowing direct localizing of various DNA sequences and physical chromosome mapping. Ribosomal DNA (rDNA) sequences belong to a family of conserved, moderately repetitive DNA, and are often used as cytogenetic markers for chromosome identification. They are organized into clusters of hundreds or thousands of gene copies separated by intergenic spacers (Roa, Guerra, 2012). Their study is used to identify patterns of chromosomal evolution, species and population differentiation, and evolutionary relationships (Rossello et al., 2022).

To date, less than half of Picea species have been analyzed by FISH (Ohri, 2021). 5S and 45S rDNA loci were mapped on chromosomes of 16 Picea species (Brown et al., 1993; Hizume, Kuzakawa, 1995; Lubaretz et al., 1996; Brown, Carlson, 1997; Hizume et al., 1999; Friesen et al., 2001; Šiljak-Yakovlev et al., 2002; Vischi et al., 2003; Sarri et al., 2008; Shibata, Hizume, 2008). The Picea-specific Sau3A family of satellite DNA was mapped on chromosomes of 11 Picea species (Shibata, Hizume, 2008). Most studies have been conducted on a small number of species from one or two localities, which is insufficient for plants with such extensive ranges as many spruce species. Furthermore, there is no unified system for classifying chromosomes in Picea karyotypes, making comparison of data obtained by different teams often problematic. It should be noted that correct identification of all homologous chromosomes is very important for comparing karyotypes and assessing phylogenetic relationships between taxa.

The aim of the present study is the comparative cytogenetic analysis of seven Picea species growing in Europe and Asia using FISH with the 5S and 45S ribosomal RNA gene probes. The intra- and interspecific karyotype diversity in the genus Picea is discussed considering the distribution of 5S and 45S rDNA loci and DAPI patterns

The 5S and 45S rDNA sites on the chromosomes of P. purpurea Masters [syn. P. likiangensis var. purpurea (Masters) Dallim. et A.B. Jacs.] and P. meyeri Rehder et E.H. Wilson naturally occurring in China were mapped here for the first time. Another species from China, P. crassifolia Komarov [syn. P. schrenkiana var. crassifolia (Komarov) Komarov and P. complanata var. crassifolia (Komarov) Gaussen], was studied from a new locality. According to E.G. Bobrov (1971) classification, P. crassifolia and P. meyeri belong to the section Picea, series Asperatae, and P. purpurea, to the section Casicta, series Likiangenses.

The study included two Eurasian species with overlapping large ranges: Norway spruce P. abies (L.) H. Karst. (section Picea, series Excelsae) and Siberian spruce P. obovata Ledeb. (section Picea, series Obovatae). Within a continuous range, the European and Siberian spruce populations gradually diverge from one species to the other. At the contact zone, these two species hybridize, forming the interspecific hybrid P. × fennica (Regel.) Kom. (Popov, 1999). The material for the study was collected from the area of “pure” species distribution.

The study also included P. schrenkiana Fisch. et C.A. Mey., section Picea, series Obovatae, which has a fairly extensive range in the mountains of Central Asia, and Serbian spruce (P. omorika (Pančić) Purkine, section Omorika) – an endemic species with an extremely limited range from Eastern Europe.

## Materials and methods

Plant material and primary cytogenetic analysis. Population seed mixtures collected from natural stands and artificial plantation were used for the study (Table 1). The seeds were germinated in Petri dishes on moist filter paper at room temperature. Root tips with a length of approximately 0.5–1.0 cm were pretreated with 0.2 % colchicine for 15–18 h at 20 °С, followed by ice water for 1 h, and then fixed in a mixture of 96 % ethanol and glacial acetic acid (3 : 1) for a minimum of 3 h.

**Table 1.**
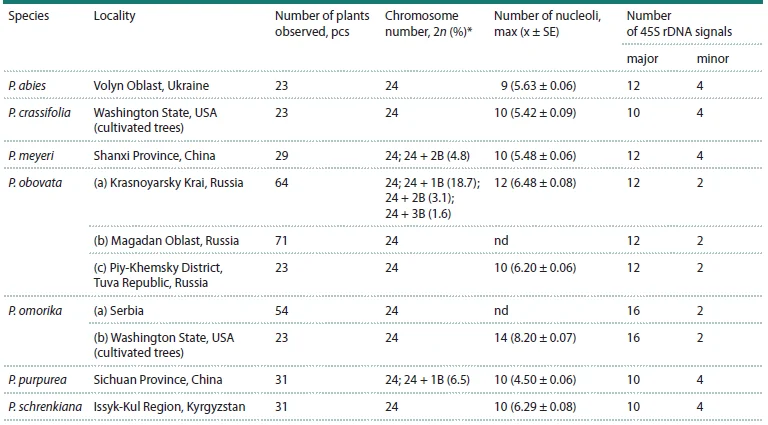
Geographic origins and cytogenetic characteristics of Picea samples * The frequency (in %) of plants with B chromosomes is indicated in parentheses.

To count the number of chromosomes, identify chromosomal and genomic abnormalities, and analyze the morphology of chromosomes, we used standard methods for conifers with our own modifications (Pravdin et al., 1972). For FISH, the roots were treated with enzymes to soften the tissue. Materials were washed in distilled water for 1 h, macerated in 0.6 % Cellulysine (Calbiochem, Switzerland) water solution, pH ~ 3–4 at 24 °С overnight or in an enzyme mix consisting of 2 % cellulase Onozuka R-10 (Sigma-Aldrich, USA) and 2 % Pectolyase Y-23 (Fisher Scientific, USA) in citrate buffer (pH 4.0) at 37 °С for 30 min.

Fluorescent in situ hybridization. Wheat DNA probe, pTa71 (Gerlach, Bedbrook, 1979), labeled with digoxygenin by nick translation was used for 45S ribosomal RNA gene mapping on P. purpurea chromosomes. The probe was detected with antibodies to anti-digoxigenin-rhodamine (Fab fragments, Sigma-Aldrich, USA). The 5S rDNA probe was amplified from genomic DNA of Larix sibirica Ledeb. Total DNA was extracted using a modified СТАВ method (Devey et al., 1996) and used as a template for PCR amplification. Two primers, P3 (5′-GAGTTCTGATGGGATCCGGTG-3′) and P4 (5′-CGCTTGGGCTAGAGCAGTAC-3′), were chosen for amplification of the entire spacer region and partial amplification of the 5S rRNA gene (Brown, Carlson, 1997; Trontin et al., 1999).

The following program was used for PCR amplification: 3 min at 96 °C, 30 cycles of (40 s at 94 °C, annealing at 53 °C for 1 min, and primer extension at 72 °C for 30 s), 10 min at 72 °C. PCR products were separated on 1 % agarose gel, stained with ethidium bromide and visualized under UV light. The fragment sizes of the 5S rDNA PCR product were approx. 250 bp. The corresponding band was excised following electrophoresis and DNA was purified using the QIAquick PCR purification kit (QIAGEN, Germany). Cloning of the 5S rDNA fragment was performed using the QIAGEN PCR Cloning Kit (QIAGEN, USA). Competent Escherichia coli XL-blue cells were used for transformation.

Recombinant colonies were selected on LB agar medium with ampicillin and Xgal/IPTG. The presence of inserts was checked by PCR with the above primers. A recombinant clone containing an insert of the expected length was selected for use as a probe for FISH. For this purpose, the cloned rDNA PCR product was labelled with Biotin-16-dUTP (Roche, Germany) by nick translation, according to the manufacturer’s protocol. The biotinylated probes were detected using avidin, conjugated with fluorescein (Fluorescein Avidin D, Vector Laboratories, USA). The hybridization signal was enhanced using fluorescein anti-avidin (Fluorescein Anti-Avidin D, Vector Laboratories, USA).

Two wheat DNA probes, pTa794 (5S rDNA) and pTa71 (45S rDNA), were used for in situ hybridization of other six Picea species studied (Gerlach, Bedbrook, 1979; Gerlach, Dyer, 1980). Fluorescent in situ hybridization was conducted as described in our previous work (Goryachkina et al., 2013)

The preparations were embedded in Vectashield mounting medium (Vector Laboratories, USA), containing 0.5 μg/ mL DAPI (4′,6-diamidino-2-phenylindole dihydrochloride, Sigma-Aldrich, USA) for chromosome staining. The chromosomes were examined with an Axio Imager D1 (Carl Zeiss, Germany) microscope and recorded with an AxioCam CCD camera using the AxioVision v. 4.6 software package. Images were processed using Adobe Photoshop CS4 (Adobe Systems, USA).

Karyotype analysis. Chromosomes were measured on 7 to 27 (for different samples) well-spread metaphase plates. The following parameters were determined for each chromosome: total length, arm ratio, position of secondary constrictions (distance from the centromere to the secondary constriction / length of the arm ×100), the presence or absence of rDNA loci, the presence or absence of DAPI positive and negative bands, the relative position of the FISH signal and DAPIbands (distance from the centromere to the middle of signal / length of the arm ×100). Pairs of homologous chromosomes were determined on the basis of chromosome morphology, the size and position of 5S and 45S rDNA loci and DAPI bands, when available. The chromosomes were numbered in order of decreasing absolute length. In the group of similar-sized metacentrics of II–VII pairs, the chromosome pairs were arranged in the same order as in the work of F. Shibata and M. Hizume (2008) to simplify the comparison of our results.

## Results

Karyotypes of all seven Picea species consist of 24 somatic chromosomes (2n = 24). Supernumerary (В) chromosomes were found in several seedlings of P. meyeri and P. purpurea; in P. obovata they were observed only in the population from Krasnoyarsky Krai (Table 1). In all species, B chromosomes were metacentric (arm ratio 1.1–1.3 %), with the length ranging from 4.05 to 4.65 μm without rDNA sites. Morphometric parameters of P. abies, P. obovata, P. omorika, P. meyeri, and P. schrenkiana chromosomes are presented in Table 2. Chromosome morphologies of P. crassifolia and P. purpurea were not measured, and for these species we provide only the position of FISH signals.

**Table 2.**
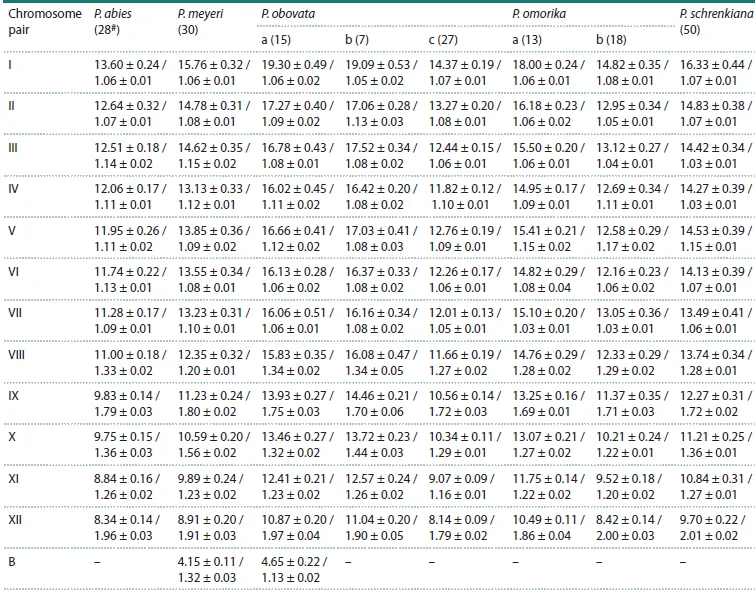
Morphometric data of the chromosome pairs of different Picea species and localities: total length / arm ratio Notе. The total length is indicated as x ± SE (μm), arm ratio is indicated as x ± SE (%); x – means, SE – standard errors; # – number of metaphases measured.

All species showed similar distribution of 5S rDNA clusters; two unequal signals were detected on both arms of a single pair of large metacentric chromosomes III (Fig. 1). A larger signal of the 5S rDNA probe was always observed in the middle of the long arm; it partially overlapped with major 45S rDNA site, but was localized closer to the centromere. A smaller, minor site was located subterminally in the opposite arm. The pTa794 probe showed more intense hybridization signals than the probe amplified from the L. sibirica genome. This was a possible reason why we were unable to identify a minor 5S rDNA site on the short arm of chromosome III in P. purpurea.

**Fig. 1.**
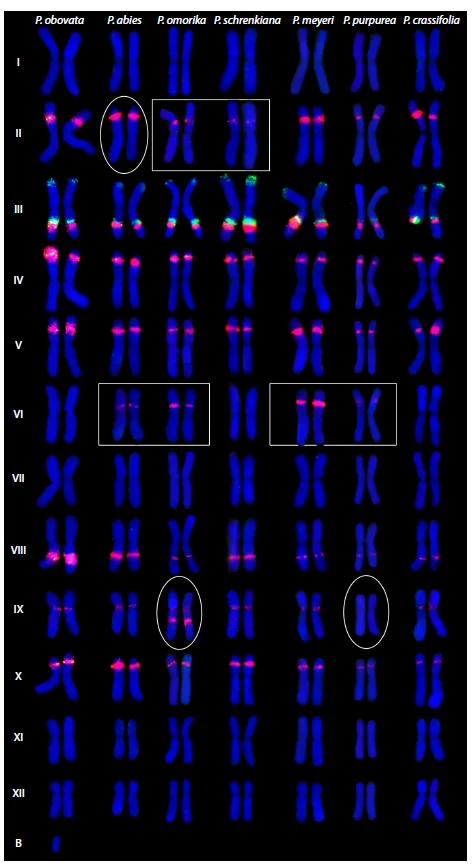
FISH mapping of the 45S rDNA (red) and 5S rDNA (green) probes on metaphase chromosomes of seven Picea species. Chromosomes of different species carrying polymorphic pTa71 signals in similar position are boxed. The chromosomes possessing unique signals or lacking any marker signals are outlined by an oval.

45S rDNA signals of varying intensity were detected on 7–9 pairs of chromosomes in the karyotypes of the studied species. Major signals were always located intercalarily and coincided with the positions of secondary constrictions. Their number varied from eight (per haploid genome) in P. omorika, six in P. abies and P. obovata, to five in P. schrenkiana, P. crassifolia, P. purpurea, and P. meyeri (Table 3, Fig. 1).

**Table 3.**
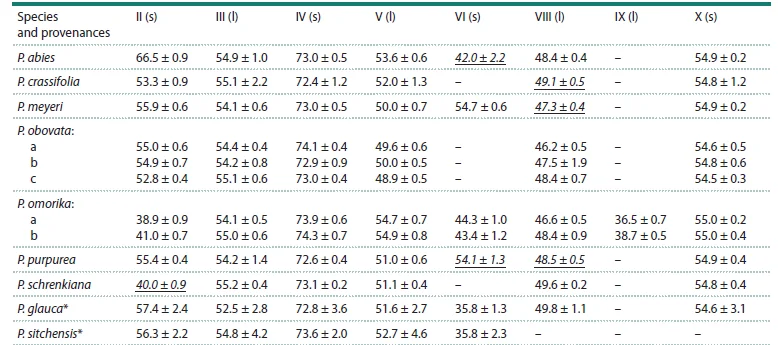
Relative position of the 45S rDNA loci (x ± SE), % on chromosomes of Picea species Notе. Minor 45s rDNA sites are given in italics and underlined. *According to (Brown, Carlson, 1997).

Interspecific differences in the localization and intensity of 45S rDNA signals were identified in chromosome pairs II, VI, VIII, IX and X. The remaining pairs of chromosomes in the studied species did not differ: chromosome pairs I, VII, XI and XII did not carry clusters of 5S and 45S rRNA ribosomal genes, and long metacentrics of pairs III, IV and V showed a similar hybridization pattern with weak variation in signal intensity.

The 45S rDNA site on the short arm of chromosome II was observed in all species, but several differences in signal location and intensity were identified (Fig. 1). As measurements showed, in P. schrenkiana and P. omorika, the 45S rDNA locus was located more proximally than in the other studied species (Table 3); in addition, hybridization signal was weaker. On chromosome VI, the 45S rDNA site was observed in four of the seven species studied, but its localization varied: in P. meyeri and P. purpurea, the signal was detected in a medial position, while in P. abies and P. omorika, it was closer to the centromere; the signal intensity also varied (Fig. 1). Chromosome pairs VIII and X carried 45S rDNA loci of similar localization (Table 3); the studied species differed in the intensity of the hybridization signal.

A minor 45S rDNA site in pericentromeric regions of large submetacentric chromosome IX was observed in six Picea species (except for P. purpurea). The arm location of this site varied depending on the species: in P. obovata and P. omorika, it was located on the long arm of chromosome IX, while in P. abies P. schrenkiana, P. crassifolia, P. meyeri, it was found on the short arm. In the karyotype of P. omorika, chromosome IX also carried a unique major 45S rDNA signal in the medial region of the long arm, coinciding with the secondary constriction.

After FISH, several intensely fluorescent DAPI-positive bands were detected in the intercalary regions of most chromosomes; rarely, the centromere region was also stained (Fig. 2). DAPI-negative bands most often corresponded to the localization regions of the ribosomal genes 5S and 45S rRNA and the centromeres of most chromosomes.

**Fig. 2.**
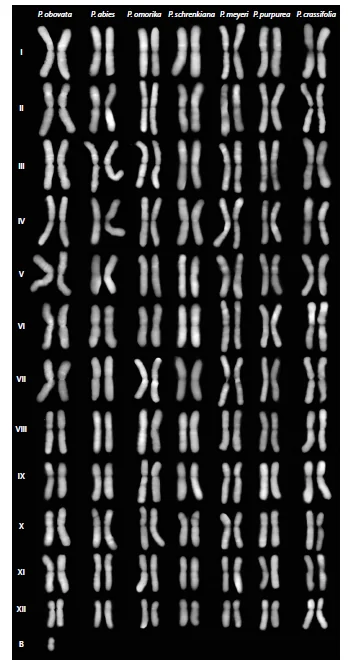
DAPI staining of metaphase chromosomes of the studied Picea species.

Permanent intercalary DAPI-positive bands with bright fluorescence appeared on chromosomes I, III and X (Fig. 2); their distribution was conservative across the species and facilitated the identification of homologous chromosomes in karyotypes. One can observe proximal positive DAPI bands on both arms of chromosome I; a double proximal and distal band on the short arm of chromosome III; and a distal band on the long arm of chromosome X.

Other DAPI-positive bands were observed irregularly on chromosomes, in some cases, only in one homologue in a pair. The intercalary DAPI bands on the short arm of chromosome II, corresponding to the 45S rDNA sites in P. obovata, P. abies, and P. meyeri, were one of the brightest. In the remaining studied species, an intercalary DAPI band was also observed on the short arm of chromosome II, but it was weaker, located closer to the centromere and did not coincide with the 45S rDNA sites. Chromosome IV in most species showed numerous thin intercalary DAPI bands on both arms. In P. obovata, P. omorika, P. meyeri and P. purpurea, bright DAPI-positive blocks were observed in the proximal region of the short arm of chromosome V. In P. omorika, P. meyeri and P. purpurea, positive DAPI bands were observed in the middle of the long arm of chromosome XII.

Molecular cytogenetic methods coupled with traditional karyological analysis based on chromosome morphologies allowed us to define pairs of homologous chromosomes in karyotypes of seven Picea species. Eight chromosome pairs, namely I, III, IV, VIII, IX, X, XI, XII, were easily identified in all species studied. Owing to similar morphological parameters of chromosomes II and V, VI and VII, it was more difficult to find their homologous pairs. Chromosomes II and V of P. obovata, P. meyeri, P. purpurea, and P. crassifolia all carried major intercalary 45S rDNA sites on short arms with similar localization (Table 3), but homologous chromosome pairs can be discriminated based on morphology and DAPI-banding. Chromosomes VI and VII of P. obovata, P. schrenkiana, P. crassifolia were difficult to distinguish because they lacked any FISH signals and had similar DAPI-patterns.

The diversity of spruce species with respect to the distribution of 5S and 45S rDNA loci and DAPI staining is reflected on a generalized idiogram of the Picea chromosomes shown on Figure 3. As can be seen, variability between species in the presence, intensity and localization of 45S rDNA signals exists for chromosomes II, VI, VIII, IX. These FISH patterns have been used to trace the relationships between Picea species, the basic premise being that more closely related species will have more similar karyotypes than distantly related ones.

**Fig. 3.**
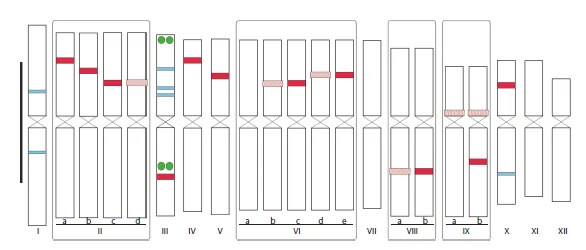
Comparative idiogram of the Picea chromosomes showing the variation of chromosomal locations of 45S rDNA. Major loci are shown as red bands, minor loci are shown as shaded red bands. 5S rDNA sites are shown as green circles; DAPI bands – as blue bands. Scale bar is 10 μm. I–XII – chromosome numbers. Chromosome II types (a) – P. abies; (b) – P. crassifolia, P. meyeri, P. obovata, P. purpurea; (c) – P. omorika; (d) – P. schrenkiana. Chromosome VI types (a) – P. schrenkiana, P. crassifolia, P. obovata; (b) – P. abies; (c) – P. omorika; (d) – P. purpurea; (e) – P. meyeri. Chromosome VIII types (a) – P. crassifolia, P. purpurea, P. meyeri; (b) – P. omorika, P. schrenkiana, P. obovata, P. abies. Chromosome IX types (a) – P. crassifolia, P. schrenkiana, P. meyeri, P. obovata, P. abies; (b) – P. omorika.

## Discussion

The genes encoding the 5S and 45S ribosomal RNA are widely used as molecular-cytogenetic markers in evolutionary and genomic studies of forest trees because of their highly conserved structure and tandem organization (Ribeiro et al., 2008). Within a species, the number of rDNA loci is generally stable, making them a convenient tool for chromosome identification. However, the number and location of sites varies between taxa, due to karyotype transformations during evolution.

Gymnosperms are known to possess one or four 5S rDNA loci per haploid genome, these sites can have both terminal and interstitial localization on the chromosome (Ribeiro et al., 2008; Garcia et al., 2017; Ohri, 2021). Much more diversity is observed in the number of 45S rDNA loci: from 2 (in most Podocarpus and Juniperus species) to 38 in Pinus taeda (Ohri, 2021). The position of 45S rDNA sites on the chromosomes of Picea and other gymnosperms is usually interstitial, whereas in angiosperms, the sites are mostly terminal (Roa, Guerra, 2012; Garcia et al., 2017).

In the Pinaceae, both ribosomal RNA gene families frequently appear on the same chromosome; many of these have both sites on the same chromosome arm. Partially overlapping 5S and 45S FISH signals are observed on Picea, Pinus and Abies chromosomes, but the Southern blot hybridization did not reveal significant cohybridization of the 26S (18S) and 5S rDNA probes in any of these. The most likely interpretation of colocalized signals is the juxtaposition of independent 5S and 45S arrays (Garcia, Kovařík, 2013). The arrangement of these signal sites was different; in some Abies species, the 5S rDNA signal is localized closer to the telomere (Shibata et al., 2004; Puizina et al., 2008), while in Picea species, the 5S rDNA signals were localized closer to the centromere. Both these 45S and 5S rDNA patterns could be recognized in different Pinus species (Liu Z. et al., 2003; Cai et al., 2006).

In the karyotypes of all studied species of Larix, and Pseudotsuga menziesii, the 5S and 45S rDNA loci were also located on the same chromosome, but on different arms (Hizume et al., 1996; Liu B. et al., 2006, 2007; Zhang et al., 2010; Goryachkina et al., 2013). Interspecific variability in the number and localization of 5S rDNA sites has been described in Pinus and Abies species (Liu Z. et al., 2003; Shibata et al., 2004; Cai et al., 2006; Puizina et al., 2008; Nkongolo, Mehes-Smith, 2012; Ohri, 2021). In the Picea and Larix species studied to date, the number of 5S rDNA loci is constant.

The chromosome carrying clusters of both 45S and 5S rDNA was found in all Picea species studied to date and therefore can be used as cytogenetic marker of spruce karyotype (Brown et al., 1993; Hizume, Kuzukawa, 1995; Brown, Carlson, 1997; Hizume et al., 1999; Šiljak-Yakovlev et al., 2002; Shibata, Hizume, 2008). A similar pattern of clustering of 5S and 45S rDNA loci on one of the long metacentrics was observed in the karyotypes of Larix species: a single 5S rDNA locus is localized terminally on the short arm of chromosome III, which also carries a major 45S rDNA site on the other arm (Liu B. et al., 2006, 2007; Zhang et al., 2010; Goryachkina et al., 2013).

The number of 45S rDNA loci on chromosomes of different Picea species has been shown to vary from 8 to 26; American and Eurasian species possess more loci than East Asian ones (Ohri, 2021). Two chromosomes, namely I and XI, did not carry clusters of 45S rDNA ribosomal genes in any spruce species studied to date. Interspecific differences were observed in the number of 45S rDNA sites and their localization on the remaining chromosomes in the karyotype. Major 45S rDNA signals coincided with the permanent secondary constrictions, indicating the functional activity of the corresponding loci, as shown in previous studies (Šiljak-Yakovlev et al., 2002).

Two loci of 45S rDNA genes were characterized by high constancy and were detected in the karyotypes of all studied Picea species: there was a medial site on the long arm of chromosome III and a subterminal site on the short arm of chromosome IV. These loci facilitated the identification of homologous chromosomes in the group of long metacentrics of pairs II–VII. The distal 45S rDNA site on the short arm of chromosome V was also quite stable; it has not been identified at present only in the North American species P. breweriana (Shibata, Hizume, 2008). In the karyotype of P. breweriana, unique major 45S rDNA sites have been found on the short arm of chromosome III and in the pericentromeric region of chromosome X; these sites have not been observed on the chromosomes of other spruce species (Shibata, Hizume, 2008). Brewer spruce has been considered to occupy an isolated position in the Omorika section of the genus Picea, and its closeness to some Asian spruce species has been confirmed by studying the nucleotide sequences of the nuclear, mitochondrial and chloroplast genomes (Ran et al., 2006, 2015; Lockwood et al., 2013).

Interesting data have been obtained by comparing the localization of polymorphic sites of 45S rDNA on chromosome pairs II, VI and VIII. These results can be considered preliminary, since to date, the 5S and 45S rRNA genes have been mapped on the chromosomes of only half of the Picea species (Brown et al., 1993; Lubaretz et al., 1996; Brown, Carlson, 1997; Hizume et al., 1999; Šiljak-Yakovlev et al., 2002; Vischi et al., 2003; Sarri et al., 2008; Shibata, Hizume, 2008). However, certain regularities that may indicate the relationship or common origin of species should be noted.

Earlier, F. Shibata and M. Hizume (2008) described two putative positions of the major 45S rDNA signal on the short arm of chromosome II: subterminal (P. abies, P. sitchensis, P. glauca) and median (P. asperata, P. crassifolia, P. jezoensis var. hondoensis, P. koraiensis, P. obovata, P. pungens var. glauca). These locations are shown in the idiogram (Fig. 3). However, G.R. Brown et al. (1993) in their original work indicated the medial localization of this signal in P. sitchensis and P. glauca (Table 3), which allows us to classify these species into the second group. So, the 45S rDNA site on chromosome II of P. abies has a unique position in the subterminal part of the short arm (Fig. 3, Table 3). According to our results, the 45S rDNA cluster on chromosome II of P. schrenkiana and P. omorika was located more proximally compared to other species, and therefore these species can be assigned to the third group (Fig. 3). Thus, the 45S rDNA locus on the short arm of chromosome II can have three variants of location in different Picea species and appears to be the most polymorphic in localization on the chromosome arm.

We found that chromosome VI could exhibit several 45S rDNA patterns, but it is present in only half of the Picea species studied to date. Among the species studied, this site had a proximal localization in P. omorika (major locus) and P. abies (minor locus). In the North American species P. glauca and P. sitchensis, major 45S rDNA loci were noted on chromosome VI, also with a proximal localization of 35.8 % (Brown, Carlson, 1997). F. Shibata and M. Hizume (2008) showed that in the karyotype of P. engelmannii, chromosome VI has a 45S rDNA signal in the proximal part of the short arm, whereas in P. koraiensis, it has a signal in the medial part of the long arm. Morphometric data showed that in P. purpurea and P. meyeri, the 45S rDNA signal on chromosome VI was located medially on the short arm, although in metacentrics, it is sometimes difficult to identify the short and long arms (Table 3). Chromosome VI in all other studied spruce species (Eurasian P. schrenkiana and P. obovata, East Asian P. crassifolia, P. asperata, P. jezoensis var. hondoensis and P. koyamai, North American P. breweriana, P. mariana and P. rubens) did not have 45S rDNA signals.

The 45S rDNA site on chromosome VIII was observed in the middle of the long arm in all studied species, but the signal intensity varied among the species. Most East Asian species (P. meyeri, P. crassifolia, P. purpurea) showed weak hybridization signals, while European and Eurasian species (P. abies, P. omorika, P. schrenkiana, P. obovata) had major 45S rDNA loci. Among the species from East Asia studied by other authors, the 45S rDNA site of similar localization on chromosome VIII was observed only in P. jezoensis var. hondoensis and was absent in P. asperata, P. koraiensis, and P. koyamai (Hizume et al., 1999; Shibata, Hizume, 2008). However, in the last three species, fluorescent staining revealed intercalary CMA+ bands on the long arm of chromosome VIII; among the East Asian species, they were not observed only in P. maximowiszii var. senanensis and P. wilsonii (Hizume, 2017). As is known, intercalary SMA+ bands can correspond to the localization regions of 45S rRNA genes.

In the species we studied, except for P. purpurea, a minor 45S rDNA site was identified in the pericentromeric region of chromosome IX. This locus was previously detected in P. abies by hybridization with the PAF1 probe containing the 45S rDNA intergenic spacer (Vischi et al., 2003). Fluorescent staining showed similar results: spruce species from North America and Eurasia contained CMA+ bands on chromosome IX, while a number of East Asian species (P. wilsonii, P. koyamae, P. maximowiczii var. senanensis, P. likiangensis) lacked these bands (Hizume, 2017). P. wilsonii, P. likiangensis and P. purpurea are shown to be phylogenetically related as can be seen from the results of recent molecular genetic analyses of the nuclear, chloroplast and mitochondrial genes (Sun et al., 2014). P. purpurea and P. likiangensis also exhibit similar morphological traits: the shape and structure of seed scales, buds and needles, color, and pubescence of their shoots (Liu T.S., 1982).

Some minor 45S rDNA sites can represent low-copy-number sites of 45S rDNA that may have been active in ancestral forms of gymnosperms and then moved to distal regions of chromosomes as a result of structural rearrangements or the activity of mobile elements (Garcia, Kovařík, 2013; Garcia et al., 2017). This phenomenon known as amphiplasty frequently occurs in allopolyploid plants (Pikaard, 2000); however, the function of “latent” loci can be restored if the existing active nucleolus organizer regions (NORs) have been lost. Thus, the minor NOR on chromosome 1R of tetraploid Triticale become functional in genotypes lucking wheat chromosomes 1B and 6B (Badaev et al., 1992). Most minor NORs do not possess true rDNA genes, but pseudogenes (Pedersen, Linde-Laursen, 1994), and they may occur due to the action of mobile elements (Dubkovsky, Dvorak, 1995; Raskina et al., 2004).

Minor pericentromeric 45S rDNA sites were common in pines; their 2n number varied from 2 to 18 in different species (Liu Z. et al., 2003; Islam-Faridi et al., 2007; Bogunić et al., 2011; Shibata et al., 2016). M. Hizume et al. (2001) isolated the 18S and 26S rDNA sequences using microdissection of pericentromeric regions of P. densiflora chromosomes and proposed that these sequences are nonfunctional because the number of nucleoli in the interphase nuclei was lesser than the number of major NORs. The minor pericentromeric 45S rDNA sites were also observed in several Larix species (Goryachkina et al., 2013).

The FISH pattern has been used to suggest relationships between the species. From this point, signal locations on chromosomes II, VI, VII, VIII, and X seem to be most informative. Close location of 45S rDNA sites had been recorded for species belonging to the same sections (or subgenera in some scientist’s opinion), established by different authors based on similarities in morphological and anatomical features (Sukachev, 1928; Dallimore, Jackson, 1966; Bobrov, 1971; Liu T.S., 1982; Schmidt, 1989; Ledig et al., 2004; Farjón, 1990).

It should be noted that intrasectional classification of the genus remains quite controversial. Furthermore, many Picea species cross readily with each other in contact ranges; cross experiments also showed hybridization between many geographically isolated spruce species, even from different continents (Wright, 1955; Mikkola, 1969). Our FISH results are largely consistent with the data of molecular phylogenetic study of the genus (Sigurgeirsson, Szmidt, 1993; Nkongolo, 1999; Ran et al., 2006, 2015; Lockwood et al., 2013).

On the other hand, similar location of 45S rDNA sites has been recorded for species from different groups of the genus Picea: P. omorika vs P. schrenkiana (the proximal 45S rDNA locus is on the short arm of chromosome II); P. omorika vs P. abies (the medial 45S rDNA is on the short arm of chromosome VI); P. meyeri vs P. purpurea (the proximal 45S rDNA is on the short arm of chromosome VI); P. omorika vs P. pungens var. glauca (the 45S rDNA is on the long arm of chromosome IX). Therefore, many aspects of taxonomic and phylogenetic relationships of Picea species are still awaiting their clarification by means of a broad range of molecular cytogenetic and genetic approaches.

## Conclusion

The combination of molecular cytogenetic methods with traditional methods of karyological analysis based on the study of chromosome morphology allows the identification of pairs of homologous chromosomes in Picea karyotypes. It was established that karyotypes of Picea species differ in number, position, and intensity of 45S rDNA signals. Similar localization of signals is usually observed in species belonging to the same sections. In some cases, similar localization of 45S rDNA sites was noted in species from different series; it requires new comparative cytogenetics and molecular genetic studies. Therefore, the method of fluorescence in situ hybridization can provide valuable information on the relationship between species and gives a new possibility for analysis of genetic diversity, evolution, intra- and interspecific variability of the genus Picea.

## Conflict of interest

The authors declare no conflict of interest.
